# Description and application of a method to quantify criterion-related cut-off values for questionnaire-based psychosocial risk assessment

**DOI:** 10.1007/s00420-020-01597-4

**Published:** 2020-11-03

**Authors:** Mathias Diebig, Peter Angerer

**Affiliations:** Institute of Occupational, Social and Environmental Medicine, Heinrich Heine University Düsseldorf, Universitätsstr. 1, 40225 Düsseldorf, Germany

**Keywords:** Psychosocial risk assessment, Psychosocial factors, Depression, Workplace risk assessment, Occupational safety and health

## Abstract

**Purpose:**

The psychosocial risk assessment is a systematic intervention process for organizations that aims at improving psychosocial working conditions as well as employee health. Based on a screening of working conditions, interventions to reduce risk factors are implemented and evaluated. What is missing for most screening instruments however are cut-off values to categorize working conditions into uncritical vs. critical, whereas the latter indicates an elevated risk for illness. To estimate and evaluate cut-off values, two studies were conducted using the receiver operating characteristic (ROC) analysis.

**Methods:**

In Study 1, a sample of 229 participants answered an online survey on depression (PHQ) and psychosocial working conditions using a questionnaire (DYNAMIK) that covers five factors important to workers' health: workload, boundary permeability, participation, leader support, and usability. Using the ROC analysis, criterion-related cut-off values were generated to predict depressive symptoms. In Study 2, these cut-off values were used to classify working conditions in the two categories of ‘critical’ and ‘uncritical’ in an independent sample (*N* = 295). It was tested for differences in the results of the two groups concerning the direct criterion of depressive symptoms and the indirect criterion of effort-reward imbalance.

**Results:**

In Study 1, cut-off values differed between the five scales and showed different values for sensitivity and specificity. In Study 2, participants exposed to critical working conditions reported more depressive symptoms as well as an effort-reward imbalance.

**Conclusions:**

Cut-off values are useful to identify working conditions as either critical or uncritical. This knowledge is important when deciding which working conditions should be optimized within the context of psychosocial risk assessment.

## Introduction

The psychosocial risk assessment is a systematic intervention process for organizations that is aimed at improving psychosocial working conditions and thus employee health and well-being (Rick and Briner 2000). The psychosocial risk assessment is grounded in various national policies and is an internationally acknowledged method that encourages organizations to design health-promoting working conditions (Leka et al. 2015). In general, psychosocial risk assessment is a multi-stage process with phases of preparation, screening, action-planning, implementation, and evaluation (Nielsen et al. 2010). In the preparation phase, the process of psychosocial risk assessment is arranged within the organization by defining fields of occupational activity, a timeline, aims, and procedures. In the screening phase, psychometric measures—mainly questionnaire based—are used to analyze psychosocial risk factors within the organization. In the action-planning phase, interventions that aim at eliminating or at least reducing the identified risks are developed. These interventions should be based on a well-founded assessment of current working conditions. In the implementation and evaluation phase, the developed interventions are put into practice and finally, it is evaluated whether they are effective in reducing the observed risk. This whole process needs to be documented to fulfill national policy demands (Beck and Lenhardt 2019; Diebig et al. 2018; European Agency for Safety and Health at Work 2014).

A frequent problem within the process of psychosocial risk assessment is the determination of priority: Which interventions that optimize working conditions have the highest priority in relation to the detected risks. This means that the observed working conditions must be ranked according to their potential health risk as well as the number of people involved. In epidemiological literature, workplaces with a high psychological risk for adverse health effects are generally defined on a theoretical basis. The job demand-control model defines jobs as critical when individuals report high demands together with low control (jobstrain; Karasek 1979). High demands are classified as study-specific answers on the demand scale above the median and low control is classified as study-specific scores on the control scale below the median score (Kivimäki et al. 2018). The effort-reward imbalance model defines high-risk workplaces based on a mismatch between effort and rewards. Particularly, a threshold with an effort-reward ratio greater than one indicates effort-reward imbalance (Siegrist 1996). To conclude, existing approaches to classify working conditions as critical vs. uncritical are generally theory-driven and not empirically determined for instruments used within the psychosocial risk assessment. In addition, these classification systems summarize various aspects of working conditions and do not provide information on a more detailed dimensional level.

This potential limitation may be overcome when criterion-related cut-off values are calculated (Lehr et al. 2010). These cut-off values quantify an elevated risk for illness. Currently, these cut-off values are missing for most instruments that are used within the psychosocial risk assessment. The receiver-operating characteristic (ROC; Altman and Bland 1994; Zweig and Campbell 1993) analysis—as a method to determine empirically based cut-off values—will be illustrated using a screening instrument that was designed to be applied within the psychosocial risk assessment (Diebig et al. 2020). This instrument covers five dimensions: workload, boundary permeability, participation, leader support, and usability. These five dimensions presumably capture core stressors in modern working environments and were generated within a mixed-methods research process aimed at thoroughly identifying relevant stressors in the context of psychosocial risk assessment. It has also been shown that these stressors explain incremental validity of important health outcomes beyond traditional screening instruments for the job demand-control model or the effort-reward imbalance model (Diebig et al. 2020).

The criterion of depression will be taken as an example to demonstrate the procedure of determining cut-off values. This criterion is important in this context because depression may be a consequence of stressful working conditions and is a highly prevalent mental illness in modern society. The average prevalence of depression in Europe is estimated at 6.6% (Hapke et al. 2019). Also, depression constitutes one of the main causes for disability-adjusted life years (Lopez et al. 2006) and is highly comorbid with other severe physical diseases like coronary heart disease (cf. Barth et al. 2004). In addition, Follmer and Jones (2018) describe that depression can be seen as one major challenge for organizations as it directly (via health care) and indirectly (via productivity loss) contributes to organizational success. In summary, it can be said that establishing prevention measures against depression is of high relevance for organizations and leads to a measurable return on investment for them (Follmer and Jones 2018).

**Fig. 1:**
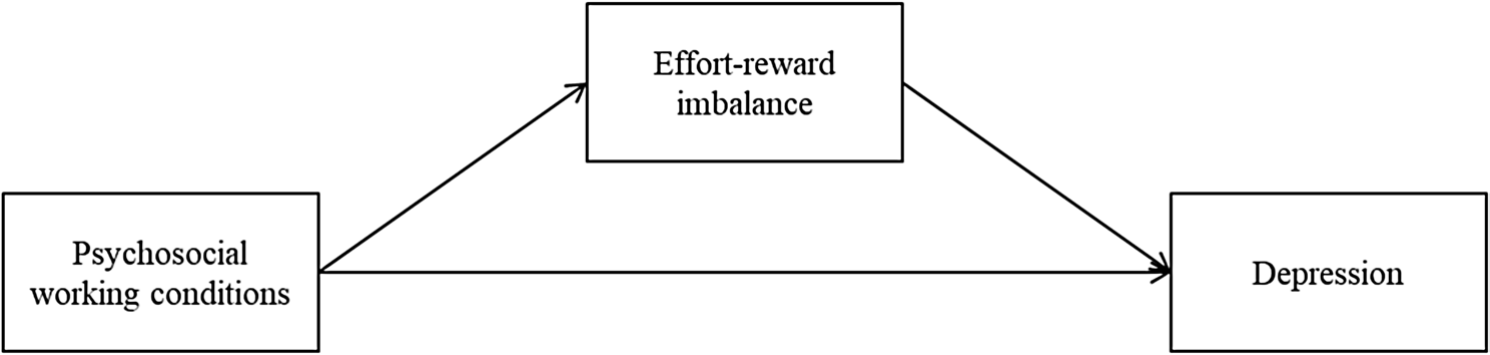
Proposed relations among study variables

We use effort-reward imbalance as a second important health outcome in our study to evaluate cut-off values. The combination of high effort and low reward has been shown to be relevant for several indicators of health in the working context (Dragano et al. 2017; Eddy et al. 2016, 2017; Kivimäki et al. 2006)—in particular when predicting depressive symptoms (Juvani et al. 2018; Rugulies et al. 2017). Our research model covers direct links between our five dimensions of adverse psychosocial work characteristics with depression as well as an indirect relation via effort-reward imbalance. Therefore, we relate psychosocial working conditions to depressive symptoms via the interpretation of working conditions. This complies with findings that psychosocial stressors exert their effects on health through intervening processes (Ganster and Rosen 2013). We define effort-reward imbalance as an important proxy when detecting depressive symptoms (cf. Fig. 1). This is important since in an organizational context it might be difficult to directly screen for depressive mood.

In sum, the aim of the present study is to apply the ROC analysis within the context of psychosocial risk assessment to quantify the clinical relevance of a questionnaire-based screening of psychosocial working conditions. This way, additional knowledge on the core question concerning which level of risk indicates a high priority of work design measures can be gained. By reporting two independent studies, we estimate cut-off values for our screening instrument (Study 1) and evaluate cut-off values based on two important health outcomes within an independent sample (Study 2).

## Background

### The DYNAMIK questionnaire to measure psychosocial working conditions

To demonstrate the procedure of developing cut-off values, we use the DYNAMIK questionnaire (Diebig et al. 2020), that covers five aspects of modern working conditions: workload, boundary permeability, participation, leader support, and usability. The questionnaire was based on a qualitative interview phase to identify specific stressors of modern working environments and was validated in four samples.

The questionnaire covers workload that results from time pressure, the interruption of work, multi-tasking, and flexibility requirements. Boundary permeability is defined as extensive overtime, insufficient breaks, work-family imbalance, and work during leisure time. Participation is defined as participation in decision-making, the influence on work content, and the influence on work methods as well as procedures. The dimension of leader support subsumes conflict with the leader, direct support by the leader, and recognition of work performance by the leader. Usability comprises technical problems and low usability of software necessary to conduct one’s work. For these five scales relations with important outcomes of health, like stress and burnout, have been demonstrated. It has been shown that all scales of the DYNAMIK questionnaire enable a thorough screening of important stressors within the psychosocial risk assessment (Diebig et al. 2020).

### Depression and psychosocial working conditions

In the previous section, it was outlined why depression is a relevant aspect for organizations. From a management perspective, the main argument is that individuals who suffer from depression are not able to fulfill their duties at work, ultimately resulting in elevated costs and productivity losses for organizations (Follmer and Jones 2018). We outlined that organizations could, therefore, benefit from the process of psychosocial risk assessment to design healthy working conditions for their employees. This way they could reduce one potential risk factor that might affect the onset of the clinical relevant depression of employees (Stansfeld and Candy 2006). From an empirical perspective, there is robust meta-analytical evidence that links psychosocial working conditions to depressive symptoms. For example, Stansfeld and Candy (2006) have shown strong associations between depression and the combination of high effort and low reward as defined in the effort-reward imbalance model (Siegrist 1996). Other meta-analyses replicated these findings. Rugulies et al. (2017) revealed that an imbalance between these two aspects of work predicts the risk of onset of depressive disorders. To sum up, findings describing the link between depression and psychosocial work characteristics—in particular effort-reward imbalance—were replicated in various meta-analyses. Recent studies extend these findings by linking other psychosocial working conditions like organizational injustice (Bonde 2008), conflicts that arise from the interplay between work and family (Amstad et al. 2011), or role ambiguity and conflict at work to depression (Schmidt et al. 2014). In sum, depression is an eligible reference criterion in the context of psychosocial risk assessment as depression is strongly related to aspects of work and has not only the high individual but also organizational relevance.

We posit a direct link between psychosocial working conditions and depression on the one hand and an indirect link through the combination of effort-reward imbalance on the other hand (cf. Fig. 1). This model guides our methodological procedure as we first develop cut-offs for the DYNAMIK questionnaire and then test to what extend these cut-off values help to differentiate between depressed and non-depressed individuals (direct link) as well as between individuals with an effort-reward imbalance (indirect link).

### Receiver-operating characteristic analysis

The ROC analysis is a widely recognized method to estimate cut-off values for several test situations and has been applied in various medical disciplines ranging from radiology (Eng 2005) to psychiatry (Behar et al. 2003). The aim of the ROC analysis is to determine the diagnostic accuracy of a diagnostic test (Altman and Bland 1994). To determine this accuracy, sensitivity and specificity are contrasted within the so-called ROC curve. Sensitivity is defined as the rate at which a test is accurately detecting an actual risk (i.e., depression) through a positive test result. Specificity is defined as the rate at which a test is identifying the absence of a risk through a negative test result (Behar et al. 2003; Lehr et al. 2010). Generally, the ROC curve indicates a test’s ability to distinguish between two conditions (risk vs. absence of the risk). Within ROC analysis, sensitivity and specificity are calculated for each potential cut-off value to detect the optimal cut-off point for a diagnostic test that is indicated by the Youden index. The Youden index is calculated as a linear combination of sensitivity and specificity $\left( {Y = {\text{sensitivity}} + {\text{specificity}} - 1} \right)$. The area under the curve (AUC) provides further information about the overall ability of a test to differentiate between depressed and non-depressed individuals. AUC values between 0.5 and 0.7 are considered to be of low diagnostic value, values between 0.7 and 0.9 of medium, and between 0.9 and 1 of high diagnostic value (Swets 1988).

## Study 1

The goal of Study 1 was to generate criterion-related cut-off values for an established measurement instrument within the psychosocial risk assessment using depression as a criterion variable. Therefore, cut-off values were determined by the ROC analysis to detect the optimal ratio of sensitivity and specificity using the Youden index.

### Method

#### Sample and Procedure

Through professional online networks and social media (e.g. xing.com) in Germany, an online survey was circulated to people working in different organizations and areas of work. The participation was voluntary. Participants were asked to answer a questionnaire for the psychosocial risk assessment and one for the measurement of depressive symptoms. In total, 229 participants completed the survey.

Of the 229 participants, 52% were female with a mean age of 35.46 years (SD = 12.89). Most of the participants worked in sectors of human health and social work activities (30%), manufacturing (9%), education (8%), other service activities (8%), information and communication (7%), as well as public administration (6%). With regard to participants’ educational background, 17% had at least completed vocational training, 12% technical college, 13% held a polytechnic degree, 35% a university degree, and 6% a doctorate. An overview of sample characteristics is given in Table 1.

**Table 1:** Overview of demographic variables and constructs collected in each study

Sample	Study 1	Study 2
*N*	229	295
Gender M/W	42%/52%	71%/19%
Age (SD)	35.46 (12.89)	47.62 (8.73)
*Sector*		
Human health and social work activities manufacturing Education Other service activities Information and communication Public administration Professional, scientific and technical activities Arts, entertainment and recreation	30% 9% 8% 8% 7% 6% 4% 3%	– 27% – – 73% – – –
*Graduation*		
Vocational training Technical college Polytechnic degree University degree Doctorate	17% 12% 13% 35% 6%	34% 15% 19% 23% 3%
*Instruments*—*Cronbach’s* *α*		
DYNAMIK workload DYNAMIK boundary permeability DYNAMIK participation DYNAMIK leader support DYNAMIK usability Depression ERI-effort	0.77 0.70 0.78 0.79 0.61 0.85 –	0.79 0.75 0.73 0.82 0.61 0.80 0.69

Due to missing values, the percentages cannot always be summed up to 100%. In Study 1 depression was measured with the PHQ-9 and in Study 2 depression was measured with the PHQ-2

The project has been approved by the ethics committee at the Medical Faculty of Heinrich Heine University Düsseldorf (no. 5562) and has been performed in accordance with the ethical standards as laid down in the 2013 Declaration of Helsinki. All steps of the study including data collection, analyses, and interpretation contributing to this work comply with the ethical standards of the relevant national and institutional committee on human experimentation. All participants submitted written informed consent.

### Instruments

The questionnaires were presented online. Participants filled in the DYNAMIK (Diebig et al. 2020) questionnaire and the Patient Health Questionnaire (PHQ-9; Kroenke et al. 2001).

*DYNAMIK* The DYNAMIK questionnaire consists of five scales with 16 items representing different stressors in the working context: workload (4 items), boundary permeability (4 items), participation (3 items), leader support (3 items), and usability (2 items). Items were recoded so that a high value represented a high level of the corresponding stressor. All items were measured on a five-point scale with a response format of 1 (never/hardly ever), 2 (seldom), 3 (sometimes), 4 (often), and 5 (always) or alternatively ranging from 1 (I strongly disagree), 2 (disagree), 3 (neutral), 4 (agree) to 5 (I strongly agree) depending on the content of the item. In addition, participants could choose “no response” if they neither wanted nor were able to give any meaningful answer. For each scale, a sum score was computed. Sample items are “I am unintentionally interrupted in my work” to measure workload or “The computer programs that I work with are easy to use” to measure software usability. Cronbach’s *α* was 0.77 for workload, 0.70 for boundary permeability, 0.78 for participation, 0.79 for leader support, and 0.61 for usability.

*Depression* The nine items of the Patient Health Questionnaire (PHQ-9) were used to measure depression (Kroenke et al. 2001, 2010; Löwe et al. 2004). The PHQ-9 is a brief measure of depressive symptom severity. Participants were asked how often they had been suffering from nine depressive symptoms over the last two weeks (sample “Little interest or pleasure in doing things”). Response format ranged from 0 (not at all), 1 (several days), 2 (more than half the days), to 3 (nearly every day). A sum score was computed, and missing values were replaced by mean values of the scale. PHQ-9 scores ranged from 0 to 27 with cut points of 5, 10, 15, and 20 representing mild, moderate, moderately severe, and severe levels of depressive symptoms (Kroenke et al. 2001, 2010). While mild and moderate values represent the normal state of individuals, moderately severe and severe levels correspond to a more critical condition. Cronbach’s *α* was 0.85.

#### Statistical analysis

The ROC analysis was computed with the package pROC (Robin et al. 2011) for the statistical software R (R Core Team 2018). The five scales of the DYNAMIK questionnaire were used as a measurement tool for the psychosocial risk assessment. Depression measured with the PHQ-9 was used as a criterion variable. For each of the five scales, sensitivity, specificity, and the Youden index were computed.

## Results and discussion

### Descriptive statistics

Descriptive statistics for study variables are presented in the following. For workload, mean value was 14.98 (*SD* = 2.70) with a minimum of 8 and a maximum of 20 (potential range of the scale was between 4 and 20). For boundary permeability, mean was 10.94 (SD = 3.30, min = 4, max = 20). Potential range of the scale was also between 4 and 20. For participation, observed values ranged between 3 and 15 (potential range also between 3 and 15) with a mean of 10.42 (SD = 2.79). For leader support, the mean was 10.55 (SD = 2.47) with observed values ranging between 3 and 15 (potential range also between 3 and 15). For usability, the mean value was 6.88 (SD = 1.39, min = 3, max = 10) with a potential range between 2 and 10.

Values of the PHQ-9 ranged between 0 and 22 in our sample with a mean of 6.09 (*SD* = 4.40). Of the participants, 40% were classified as having only mild and 42% only moderate levels of depressive symptoms, while 12% reported having moderately severe, and 6% severe levels of depressive symptoms. Observed prevalence rates are higher than prevalence rates of a representative German sample for the mild (40% vs. 76%), moderate (42% vs. 18%), moderately severe (12% vs. 4.3%), as well as the severe (6% vs. 1.3%) levels of depressive symptoms (Kocalevent et al. 2013).

### Receiver-operating characteristic analysis

First, participants with mild and moderate depressive symptoms were grouped into the category ‘non-depressed’ (82% of participants). Participants with moderately severe and severe levels of depressive symptoms were grouped into the category ‘depressed’ (18% of participants). Second, we conducted ROC analysis to evaluate the diagnostic power of the DYNAMIK scales. The presence or absence of depression served as a classification variable. Results of ROC analysis are presented in Table 2.

**Table 2:** Sensitivity, specificity, and Youden index for different cut-off points for DYNAMIK scales

Cut-off	Sensitivity	CI 95%	Specificity	CI 95%	Youden index	CI 95%
*Workload*						
15.5	0.60	0.45–0.76	0.55	0.47–0.62	1.16	0.98–1.34
16.5	0.39	0.24–0.55	0.74	0.68–0.81	1.14	0.97–1.32
17.5	0.32	0.16–0.47	0.89	0.83–0.93	1.20	1.05–1.36
18.5	0.26	0.13–0.42	0.94	0.90–0.97	1.20	1.06–1.35
19.5	0.10	0.03–0.21	0.96	0.93–0.98	1.06	0.97–1.18
*Boundary permeability*						
8.5	10.00	10.00–10.00	0.29	0.23–0.36	1.29	1.23–1.36
9.5	0.92	0.81–10.00	0.40	0.33–0.48	1.33	1.20–1.44
10.5	0.92	0.81–10.00	0.50	0.43–0.57	1.42	1.30–1.53
11.5	0.76	0.63–0.89	0.63	0.57–0.70	1.40	1.24–1.55
12.5	0.58	0.42–0.74	0.73	0.67–0.79	1.31	1.14–1.48
*Participation*						
7.5	0.37	0.21–0.53	0.87	0.82–0.91	1.24	1.07–1.40
8.5	0.47	0.31–0.63	0.78	0.72–0.84	1.26	1.09–1.43
9.5	0.63	0.47–0.79	0.71	0.64–0.78	1.34	1.17–1.50
10.5	0.74	0.60–0.87	0.54	0.46–0.61	1.28	1.12–1.43
11.5	0.87	0.76–0.97	0.43	0.35–0.50	1.29	1.17–1.42
*Leader support*						
9.5	0.55	0.39–0.71	0.73	0.66–0.79	1.28	1.12–1.44
10.5	0.66	0.50–0.79	0.63	0.56–0.70	1.28	1.13–1.45
11.5	0.92	0.81–10.00	0.41	0.35–0.49	1.34	1.22–1.44
12.5	0.95	0.87–10.00	0.24	0.18–0.31	1.19	1.09–1.27
13.5	0.95	0.87–10.00	0.14	0.08–0.19	1.08	0.98–1.16
*Usability*						
5.5	0.76	0.63–0.89	0.14	0.09–0.19	0.90	0.76–1.04
6.5	0.58	0.42–0.74	0.32	0.25–0.39	0.90	0.74–1.07
7.5	0.37	0.24–0.53	0.69	0.63–0.76	1.06	0.90–1.23
8.5	0.05	0.00–0.13	0.88	0.83–0.93	0.93	0.85–1.02
9.5	0.00	0.00–0.00	0.99	0.98–10.00	0.99	0.98–1.00

*CI* confidence intervall

Cut-off values differ between the five scales. For workload, the optimal cut-off was 17.5 with a sensitivity of 0.32 (95% CI [0.16 0.47]), a specificity of 0.89 (95% CI [0.83.93]), and a Youden index of 1.20 (95% CI [1.05 1.36]). The *AUC* for workload was 0.61 (95% CI [0.51 0.72]). For boundary permeability, the optimal cut-off was 10.5 with a sensitivity of 0.92 (95% CI [0.81 1.00]), a specificity of 0.50 (95% CI [0.43 0.57]), and a Youden index of 1.42 (95% CI [1.30 1.53]). The *AUC* for boundary permeability was 0.74 (95% CI [0.67 0.81]). For participation, the optimal cut-off was 9.5 with a sensitivity of 0.63 (95% CI [0.47 0.79]), a specificity of 0.71 (95% CI [0.64 0.78]), and a Youden index of 1.34 (95% CI [1.17 1.50]). The *AUC* for participation was 0.72 (95% CI [0.64 0.81]). For leader support, the optimal cut-off was 11.5 with a sensitivity of 0.92 (95% CI [0.81 1.00]), a specificity of 0.41 (95% CI [0.35 0.49]), and a Youden index of 1.34 (95% CI [1.22 1.44]). The *AUC* for leader support was 0.72 (95% CI [0.63 0.81]). For usability, the optimal cut off was 7.5 with a sensitivity of 0.37 (95% CI [0.24 0.53]), a specificity of 0.69 (95% CI [0.63 0.76]), and a Youden index of 1.06 (95% CI [0.90 1.23]). The *AUC* for usability was 0.46 (95% CI [0.36 0.57]).

## Study 2

The aim of Study 2 was to evaluate the empirically generated cut-off values of Study 1 within an independent sample. It was tested whether there were differences in the reported level of depression and effort-reward imbalance with participants working under conditions that were classified as ‘critical’ in comparison to participants who fall under the category of ‘uncritical’ working conditions.

### Method

#### Sample and procedure

In Study 2, participants were recruited out of seven companies in Germany that enrolled in a research project and agreed to participate in the process of psychosocial risk assessment. The psychosocial risk assessment was completed following the structured phases of preparation, screening, action planning, implementation, and evaluation. The screening was operationalized with the DYNAMIK questionnaire to assess psychosocial working conditions. For the members of the seven companies, participation was voluntary, and participants had to give informed consent before taking part in the study. Again, the university’s institutional ethic committee approved the study.

In total, 295 employees from the seven companies took part in the psychosocial risk assessment and gave information on demographics, psychosocial working conditions, depressive symptoms, and effort-reward imbalance. Mean age of the 295 participants was 47.62 years (SD = 8.73) and 71% were male. Of the participants, 73% worked in the information and communication sector and 27% in manufacturing. Concerning the highest educational degree, 34% of the participants completed vocational training, 15% technical college, 19% achieved a polytechnic degree, 23% a university degree, and 3% a doctorate.

#### Instruments

The DYNAMIK questionnaire (Diebig et al. 2020) was administered to screen psychosocial working conditions, the PHQ-2 (Kroenke et al. 2003) to screen for depression, and the effort–reward imbalance (ERI) questionnaire (Siegrist et al. 2009) to screen for an imbalance between effort and reward.

*DYNAMIK* Similarly to Study 1, participants answered the 16 items of the DYNAMIK questionnaire that represent workload, boundary permeability, participation, leader support, and usability. Cut-off values derived from Study 1 were applied to classify working conditions as critical vs. uncritical: 17.5 for workload, 10.5 for boundary permeability, 9.5 for participation, 11.5 for leader support, and 7.5 for usability. Cronbach’s *α* was 0.79 for workload, 0.75 for boundary permeability, 0.73 for participation, 0.82 for leader support, and 0.61 for usability.

*ERI* The short version of the ERI questionnaire (Siegrist et al. 2009) was used as a screening instrument for effort-reward imbalance. The questionnaire consisted of 10 items in total representing an effort score (3 items; e.g., “I have constant time pressure due to a heavy workload”) and a reward score (7 items, e.g., “I receive the respect I deserve from my superior or a respective relevant person”). Items were answered on a five-point scale ranging from 1 (I strongly disagree) to 5 (I strongly agree). Average scores were calculated, and an effort-reward ratio was computed by dividing the effort score by the reward score. Cronbach’s *α* for the effort was 0.69 and 0.82 for a reward.

*Depression* The two items of the Patient Health Questionnaire (PHQ-2) were used to measure depression (Kroenke et al. 2003; Manea et al. 2016). The PHQ-2 asks how often the participants have been suffering from depressed mood and anhedonia (loss of interest) in the last two weeks. Items were answered on a four-point scale ranging from 0 (not at all) to 3 (nearly every day). A sum score was computed for each participant, and missing values were replaced by mean values of the scale. Cronbach’s *α* was 0.80.

#### Statistical analysis

To compare means in outcome variables based on the classification of working conditions, independent t-tests were calculated using IBM SPSS Statistics 25. Cohen’s *d* (Cohen 1988) was computed as a measure of effect size for groups with different group sizes with $d=\frac{{M}_{1}- {M}_{2}}{{S}_{\mathrm{p}}}$. *M*_1_ and *M*_2_ represent means of both groups and *S*_p_ represents the pooled standard deviation of both groups with ${S}_{\mathrm{p}}=\sqrt{\frac{\left({N}_{1}-1\right)*{S}_{1}^{2}+\left({N}_{2}-1\right)* {S}_{2}^{2}}{{N}_{1}+ {N}_{2}-2}}$. According to Cohen (1988), an effect size between 0.20 and 0.50 represents a small effect, a value between 0.50 and 0.80 a medium effect, and a value above 0.80 a large effect.

### Results

We conducted *t* tests for independent samples to compare the mean values of the two groups that were classified based on the respective cut-off value for each scale (cf. Table 3). With regard to workload, both groups did not significantly differ regarding the PHQ-2 scores, *t*(291) = − 1.49, *ns*. Regarding the ERI, the critical group significantly differed from the uncritical group [*t*(293) = 4.88, *p* < 0.01] with an effect size of Cohen’s *d* = 0.95.

**Table 3:** Results of *t* tests for independent samples to test for differences between groups

	*M*_critical_	*n*_critical_	SD_critical_	*M*_uncritical_	*n*_uncritical_	SD_uncritical_	*t*	*df*	Cohens *d*	*p* value
*Dependent variable: effort-reward imbalance*										
Workload	1.62	29	0.55	1.20	266	0.43	4.88	293	0.95	0.000
Boundary permeability	1.52	97	0.52	1.10	198	0.35	7.10	140	1.00	0.000
Participation	1.52	50	0.59	1.18	245	0.41	4.84	293	0.75	0.000
Leader support	2.09	10	0.82	1.21	285	0.41	3.37	9	2.05	0.008
Usability	1.53	12	0.65	1.23	283	0.44	2.27	293	0.67	0.138
*Dependent variable: depression*										
Workload	2.11	28	1.47	1.66	265	1.50	1.49	291	0.30	0.138
Boundary permeability	2.28	96	1.56	1.43	197	1.39	4.74	291	0.59	0.000
Participation	2.82	50	1.79	1.48	243	1.33	5.02	60	0.95	0.000
Leader support	4.30	10	1.70	1.61	283	1.41	5.86	291	1.89	0.000
Usability	3.00	12	1.76	1.65	281	1.47	3.09	291	0.91	0.002

*M* mean; *n* group size, *SD* standard deviation, *t* test statistic of the *t*-test, *df* degrees of freedom of the t test

For boundary permeability, both groups differed with regard to PHQ-2 scores [*t*(291) = 4.74, *p* < 0.01]. Cohen’s *d* was 0.59. With regard to ERI values, the critical group significantly differed from the uncritical group [*t*(140) = 7.10, *p* < 0.01, Cohen’s *d* = 1.00].

For participation, mean PHQ-2 score for the critical group was higher than for the uncritical group [*t*(60) = 5.02, *p* < 0.01, Cohen’s *d* = 0.95]. The ERI score of the critical group was also significantly higher than mean value for the uncritical group [*t*(293) = 4.84, *p* < 0.01] with Cohen’s *d* = 0.75.

For leader support, the critical group scored higher in the PHQ than the uncritical group [*t*(291) = 5.86, *p* < 0.01, Cohen’s *d* = 1.89]. For the ERI score, the critical group’s mean was significantly higher than the uncritical group’s mean [*t*(9) = 2.05, *p* < 0.01, Cohen’s *d* = 2.05].

With regard to usability, both groups significantly differed [*t*(291) = 3.09, *p* < 0.01, Cohen’s *d* = 0.91] in PHQ-2 scores but not in ERI-scores [*t*(293) = 2.27, *p* < 0.01, Cohen’s *d* = 0.67, ns]. In sum, cut-off values determined in Study 1 enabled a valid classification of participants in Study 2. Overall, groups differed regarding their level of depression as well as effort-reward imbalance.

## General discussion

To summarize, the main goal of the study was to compute criterion-related cut-off values for five central stressors within the psychosocial risk assessment via ROC analysis. We applied this method and focused on depression as a relevant outcome criterion in occupational health. In the second step, cut-off values were evaluated using an independent sample. Results showed that cut-off values tend to be a promising approach to classify working conditions in the two broad categories of ‘critical’ and ‘uncritical’. Participants exposed to critical working conditions in the domains of boundary permeability, participation, leader support, and usability reported more depressive symptoms as well as a greater effort-reward imbalance.

Study 1 shows the development of different cut-off values for each of the five scales of the DYNAMIK questionnaire. For each scale, the Youden index varied in its level. Some scales displayed a high level of both aspects of diagnostic validity (sensitivity and specificity) while other scales only displayed high levels for one of these aspects. A potential explanation for these results might be that each scale has a different relative weight in explaining variance within the criterion depression (Diebig et al. 2020). In addition, some scales have a higher sum score than others, which indicates that certain aspects of work—for instance workload—are more prevalent. In this case, a very high value of this stressor is needed to differentiate between depressed and non-depressed individuals.

In Study 2, cut-off values were evaluated. Here, most groups classified by cut-off values also differed with regard to the evaluation criteria depression and imbalance between effort and reward. Yet, for workload, groups did not differ regarding depressive symptoms. Also, for usability, groups did not differ with regard to their perceived level of effort-reward imbalance. Similarly to the results of Study 1, it might be that both variables only have small predictive validity concerning the evaluation criteria. Therefore, calculated cut-off values for these variables do not provide enough information to classify working conditions.

Generally, procedures that facilitate the labeling of critical and uncritical working conditions focus on two different approaches: theoretical approaches and benchmarks. The ERI model, as an example of a theoretical approach, postulates that an imbalance between effort and reward is most relevant for individuals’ health (Siegrist et al. 2004). In that case, a ratio between effort and reward greater one indicates unfavorable working conditions with effort exceeding reward. This approach has been challenged by empirical research (Lehr et al. 2010) showing that lower cut-off values perform better in predicting health problems. Secondly and with reference to the benchmark approach, some questionnaires compare observed values of psychosocial working conditions with suitable values observed in other samples from organizations of the same sector. Yet, this approach only reflects a comparison between a current organization and a comparable one ignoring potential differences between organizations that may serve as main drivers in explaining health effects. To conclude, both approaches have some limitations in classifying observed values as critical or uncritical when they are above or below the mean value of a comparison group. This limitation can be overcome by focusing on empirically generated cut-off values using the ROC analysis.

This study wants to encourage researchers to empirically determine cut-off values for screening instruments within the psychosocial risk assessment. This procedure can help detect which working conditions need to be improved to create a healthier working environment.

### Practical implications

The procedure outlined in this study aims at presenting a method that helps to generate information for the process of psychosocial risk assessment. It serves as a guideline to determine interventions with higher priorities in relation to detected risks. Therefore, cut-off values for a screening instrument were developed based on the Youden index aimed at identifying optimal cut-offs as a function of sensitivity as well as specificity. Cut-off values varied depending on the underlying dimension of the screening instrument and thus represent the different impact they have on the outcome of depression. Practitioners may use this information to decide which working conditions need improvement to prevent depression of their employees. This information helps to design healthier working conditions and to focus on those aspects of work that pose a high risk for potential unfavorable health outcomes. With this, we encourage researchers to apply the ROC-analysis to generate empirically driven cut-offs for screening instruments that are applied within the psychosocial risk assessment.

### Limitations and future directions

Apart from the study’s several strengths like the development and evaluation of the cut-off values within two independent samples, some limitations have to be mentioned as well. First, due to limitations of the overall length of questionnaires applied, only short forms of validated measures could be implemented in this study. Particularly in Study 1, the PHQ-9 was used whereas in Study 2 only the shorter version, the PHQ-2, could be implemented. Yet, recent research has demonstrated that the short versions of these two questionnaires are strongly related to the corresponding full versions and also significantly correlate with each other (Manea et al. 2016). Future research should combine the use of the PHQ questionnaires together with a clinical diagnosis of depression resulting from a structured clinical interview to enable a more fine-grained assessment of clinical symptoms of depression.

Second, observed prevalence rates of depressive symptoms in our study are higher than prevalence rates reported within a representative German sample (Kocalevent et al. 2013). This finding raises questions about the representativeness of our sample that might result in restricted external validity of our results. Yet, the sample presented in Kocalevent et al. (2013) was based on a nationally representative household survey in Germany. The sample in our study, however, was recruited from the working population. Since our main research goal was to demonstrate a method to empirically derive cut-off values, we comply with Rothman et al. (2013), who argue that this kind of research question does not require representative sampling. However, it should be noted that this topic is controversially discussed in the scientific literature (Richiardi et al. 2013).

Third, it was only possible to generate data in a cross-sectional manner. According to this, only one source could be used as an informant for predictor and criterion variables (Podsakoff et al. 2003). Future studies should, therefore, aim at collecting data in a longitudinal manner and aim at measuring working conditions and health outcomes from different sources.

Fourth, the sample size for the development and validation of the cut-off values was rather small. Future studies should adopt the outlined procedure and compute cut-off values based on representative samples for different types of jobs. Following this, the sample used to generate cut-off values was not clinical in nature i.e., classifications of participants as ‘depressed’ and ‘non-depressed’ were not based on a clinical diagnosis (Lehr et al. 2010). Since the aim of the psychosocial risk assessment is to design healthy working conditions (Beck and Lenhardt 2019; Rick and Briner 2000), our focus lies on healthy individuals that form the basis of employees within an organization.

## Conclusion

In this study, the ROC analysis was presented as a method to empirically generate criterion-related cut-off values for a screening instrument within the psychosocial risk assessment. Cut-off values were identified to classify working conditions as either critical or uncritical. We would like to encourage researchers to empirically determine cut-off values for screening instruments within the psychosocial risk assessment. This procedure can help detect which working conditions need to be improved to create a healthier working environment.

## Appendix

**Table:**

Content of the DYNAMIK Questionnaire	
*Work load*	
1	WL01: time pressure (+)
2	WL02: interruption of work (+)
3	WL03: multi-tasking (+)
4	WL04: flexibility requirements (+)
*Boundary permeability*	
1	BP01: extensive overtime (+)
2	BP02: insufficient breaks (−)
3	BP03: work-family balance (+)
4	BP04: work during leisure time (+)
*Participation*	
1	PN01: participation in decision-making (+)
2	PN02: influence on work content (+)
3	PN03: influence on work methods/procedures (+)
*Leader support*	
1	LS01: conflict with the leader (−)
2	LS02: support by the leader (+)
3	LS03: recognition of work performance (+)
*Usability*	
1	USB01: technical problems (−)
2	USB02: usability (+)
