# Correction to: Description and application of a method to quantify criterion‑related cut‑off values for questionnaire‑based psychosocial risk assessment

**DOI:** 10.1007/s00420-021-01746-3

**Published:** 2021-07-30

**Authors:** Mathias Diebig, Peter Angerer

**Affiliations:** Institute of Occupational, Social and Environmental Medicine, Heinrich Heine University Düsseldorf, Universitätsstr. 1, 40225 Düsseldorf, Germany

## Correction to: International Archives of Occupational and Environmental Health (2021) 94:475–485 10.1007/s00420-020-01597-4

The article: Description and application of a method to quantify criterion‑related cut‑off values for questionnaire‑based psychosocial risk assessment, written by Mathias Diebig, Peter Angerer was originally published electronically on the publisher’s internet portal (currently SpringerLink) on 3 November 2021 without open access.

With the author(s)’ decision to opt for Open Choice the copyright of the article changed on 15 August 2021 © The Author(s) 2021 and the article is forthwith distributed under the terms of the Creative Commons Attribution 4.0 International License (http://creativecommons.org/licenses/by/4.0/), which permits use, duplication, adaptation, distribution and reproduction in any medium or format, as long as you give appropriate credit to the original author(s) and the source, provide a link to the Creative Commons license and indicate if changes were made.

**Open Access** This article is distributed under the terms of the Creative Commons Attribution 4.0 International License (http://creativecommons.org/licenses/by/4.0/), which permits unrestricted use, distribution, and reproduction in any medium, provided you give appropriate credit to the original author(s) and the source, provide a link to the Creative Commons license, and indicate if changes were made.

The original article was corrected.

